# Low-level and high-level modulations of fixational saccades and high frequency oscillatory brain activity in a visual object classification task

**DOI:** 10.3389/fpsyg.2013.00948

**Published:** 2013-12-18

**Authors:** Maciej Kosilo, Sophie M. Wuerger, Matt Craddock, Ben J. Jennings, Amelia R. Hunt, Jasna Martinovic

**Affiliations:** ^1^School of Psychology, University of AberdeenAberdeen, UK; ^2^Department of Psychology, City University LondonLondon, UK; ^3^Department of Psychological Sciences, Institute of Psychology, Health and Society, University of LiverpoolLiverpool, UK; ^4^Institute for Experimental Psychology and Methods, University of LeipzigLeipzig, Germany; ^5^Department of Ophthalmology, McGill Vision Research, McGill UniversityMontreal, QC, Canada

**Keywords:** visual object representation, parallel visual pathways, color, luminance, fixational saccades, microsaccades, EEG, gamma-band activity

## Abstract

Until recently induced gamma-band activity (GBA) was considered a neural marker of cortical object representation. However, induced GBA in the electroencephalogram (EEG) is susceptible to artifacts caused by miniature fixational saccades. Recent studies have demonstrated that fixational saccades also reflect high-level representational processes. Do high-level as opposed to low-level factors influence fixational saccades? What is the effect of these factors on artifact-free GBA? To investigate this, we conducted separate eye tracking and EEG experiments using identical designs. Participants classified line drawings as objects or non-objects. To introduce low-level differences, contours were defined along different directions in cardinal color space: S-cone-isolating, intermediate isoluminant, or a full-color stimulus, the latter containing an additional achromatic component. Prior to the classification task, object discrimination thresholds were measured and stimuli were scaled to matching suprathreshold levels for each participant. In both experiments, behavioral performance was best for full-color stimuli and worst for S-cone isolating stimuli. Saccade rates 200–700 ms after stimulus onset were modulated independently by low and high-level factors, being higher for full-color stimuli than for S-cone isolating stimuli and higher for objects. Low-amplitude evoked GBA and total GBA were observed in very few conditions, showing that paradigms with isoluminant stimuli may not be ideal for eliciting such responses. We conclude that cortical loops involved in the processing of objects are preferentially excited by stimuli that contain achromatic information. Their activation can lead to relatively early exploratory eye movements even for foveally-presented stimuli.

## Introduction

In order to acquire sufficient information from the complex and dynamically changing environment, the visual system implements various strategies. One such strategy is to perform eye movements in order to scan the visual scene, while intermittently maintaining gaze at objects of interest. The fovea is the central part of the retina with highest spatial acuity and is responsible for the acquisition of fine spatial details during fixations, making foveation an excellent strategy for acquiring visual information. Fixations themselves are dynamic events, during which different classes of small, involuntary eye movements have been recognized: these include microsaccades, drifts and tremors. Cornsweet (1956) suggested that the purpose of microsaccades is to counteract the effects of other fixational eye movements, such as tremor and drift - namely, to correct the eye position so that fixation returns to the target. Engbert and Kliegl (2004) refined Cornsweet's (1956) suggestions. Their analysis revealed that microsaccades operated on two time scales of different characteristics. On a short time scale (up to 20 ms), microsaccades increased fixation errors, thus increasing retinal image shifts. This most likely contributes to the prevention of perceptual fading (see Hubel and Wiesel, 1968). However, over longer time intervals (100–400 ms) microsaccades lead to a reduction of fixation errors so that fixation was maintained. A recent study by Mergenthaler and Engbert (2010) provided evidence for a microsaccade dichotomy of a different kind: a bimodal saccade amplitude distribution was observed when participants were asked to freely view natural scenes. Larger saccades (>0.4°) behaved differently than very small saccades (<0.4°), indicating that larger saccades during fixation could be inspection saccades rather than microsaccades. The purpose of these fixational saccades is likely to be selection or re-selection of scene attributes that are relatively close to fixation.

Saccades and microsaccades are both generally controlled by the superior colliculus (Hafed et al., 2009) which receives input directly from the retina, as well as cortical input from perceptual areas. Therefore, at the level of the superior colliculus subcortical low-level inputs converge with cortical loops that provide high-level information used for ocular control. Both bottom-up and top-down factors can modulate the rate of microsaccades (Betta and Turatto, 2006; Valsecchi et al., 2009; Laubrock et al., 2010; for reviews see Martinez-Conde et al., 2009; Rolfs, 2009; for a recent model see Engbert, 2012). In their study on low-level influences on microsaccade rates, Valsecchi and Turatto (2007) looked at microsaccadic responses to events often thought to be “invisible” to the superior colliculus since its superficial layers which receive direct retinal inputs do not support color-opponent processing (Marrocco and Li, 1977; also see White et al., 2009). Valsecchi and Turatto (2007) hypothesized that if microsaccades are generated solely by a low-level circuit involving the retina and the superior colliculus, microsaccadic rates should not be affected by the presentation of a stimulus which is isoluminant with the background. However, microsaccadic rates were very similar for both isoluminant and luminance-defined stimuli. They interpreted this as evidence that microsaccades elicited by isoluminant stimuli were driven by cortical loops. The idea that small fixational saccades can be modulated by cortical inputs was further supported by another study (Otero-Millan et al., 2008) which looked at microsaccadic responses in free-viewing and visual search tasks. In free exploration of a natural scene, the highest rates of microsaccades occurred during fixation of human faces. In the search task, large increases in microsaccade rates occurred in image regions containing identified targets. Otero-Millan et al.'s (2008) findings imply that foveation of targets is an essential determinant of microsaccadic behavior and that this is determined by high-level as well as low-level image content.

This line of research into the role of fixational saccades in object processing coincides with the findings reported by Yuval-Greenberg et al. (2008). These authors demonstrated that the brief broadband peak in the induced gamma-band frequency range in the electroencephalogram (EEG) actually reflects a peak in the rate of miniature fixational saccades. Induced GBA is high frequency (above 30 Hz) oscillatory activity which is neither time- nor phase-locked to stimulus onset, as opposed to stimulus-locked evoked activity. Until the publication of Yuval-Greenberg et al.'s (2008) study, iGBA was widely assumed to reflect a neural oscillation associated with higher-order cortical activity, including object representation, memory, attention and awareness (for more recent reviews see Tallon-Baudry, 2009; Herrmann et al., 2010; Rieder et al., 2011). However, saccades are also induced by the stimulus. Eye muscle movements associated with each saccade generate a spike in high-frequency electrical activity recorded from the scalp with EEG. Since microsaccades and induced gamma-band activity (iGBA) share similar temporal dynamics, the high-frequency output of these eye movements can be confused with a genuine cortical response. Engbert and Kliegl (2003) report a characteristic microsaccadic response after the onset of an event: the microsaccadic rate drops substantially below its normal rate, reaching a minimum at around 150 ms after event onset. This is followed by a substantial rate increase, which reaches a maximum at around 350 ms and returns to baseline level around about 500 ms after event onset. This “signature” has been consistently demonstrated in other studies in response to novel visual or auditory stimuli (for a review, see Rolfs, 2009). The timing of the broadband iGBA peak overlaps with this microsaccadic maximum, being most pronounced around 200–350 ms after the stimulus has been presented. Yuval-Greenberg et al. (2008) showed that the iGBA is time-locked to the onset of miniature saccades. However, iGBA may also coincide with microsaccades because both are triggered by similar perceptual processes (for reviews, see Melloni et al., 2009; Martinovic and Busch, 2011). Thus, iGBA is likely to contain both an artifactual, muscular component and an underlying genuine, cortically-generated oscillation. A recent study by Hassler et al. (2011) demonstrated just that: removal of the ocular artifact revealed an underlying iGBA which was still enhanced for object as opposed to non-object images.

Previous experiments on fixational saccades generally investigated low-level visual processing and its modulation by attention, while studies investigating the contribution of fixational saccades to iGBA looked at high-level vision. In this study, we aim to look at both low and high-level modulations of fixational saccades. We recorded fixational saccades using the paradigm from a previously reported EEG experiment on low and high-level factors in object classification (Martinovic et al., 2011). Since that study focused on event related potentials (ERPs), we reanalyzed its dataset to examine evoked and total GBA. Total GBA (tGBA) is a sum of both evoked and iGBA. To isolate iGBA, a common approach is to subtract the ERP from each single trial, theoretically removing evoked GBA. However, Truccolo et al. (2002) demonstrate that there is no way to remove evoked activity from the signal and be sure that what is remaining is only “induced,” as removing the ERP from each trial relies on the inaccurate assumption that the evoked signal is completely stationary. This leaves residual “evoked” signals on each trial. As substantial contributions of the evoked signal to the gamma-band are largely centered in frequencies below 40 Hz, occurring before 150–200 ms, the contribution to the GBA after 200 ms is mainly driven by the induced part (e.g., see Fründ et al., 2007).

We added several additional participants in order to increase the power for the gamma frequency-band analyses, which were reliant on the algorithm for microsaccadic artifact removal proposed by Keren et al. (2010), applied successfully in a previous study by Craddock et al. (2013). Although we collected fixational saccade and tGBA data in separate experiments with different participants, which limits how strongly we can draw conclusions on their relation to each other, we were able to compare lower and higher-level influences on fixational saccades themselves and on tGBA after artifact correction. Finally, the study also aimed to examine evoked gamma-band activity (eGBA; 30–40 Hz at approx. 50–150 ms), which can be modulated by object class under specific circumstances (Herrmann et al., 2004a; Fründ et al., 2008; for a review see Martinovic and Busch, 2011) but is also highly influenced by low-level stimulus properties (Busch et al., 2004; Fründ et al., 2007). Evoked gamma-band activity has been hypothesized to reflect a memory match and to act as a precursor to iGBA by Herrmann et al. (2004b).

Participants responded to simple line-drawings presented on the screen, indicating whether these drawings showed familiar, nameable objects or novel, unnamable images (i.e., non-objects). The lines were defined along different directions in DKL color space (Derrington et al., 1984) to differentially excite post-receptoral mechanisms that are distinguished at the level of lateral geniculate nucleus. Luminance is defined as the weighted sum of L and M cone excitation, with S-cones contributing only at high levels of overall luminance (Ripamonti et al., 2009). The cone-opponent mechanisms process either the weighted difference between L and M cone excitation (L − M) or the weighted difference between S-cone excitation and a sum of L and M cone excitation [S − (L + M)]. These mechanisms roughly map onto the three visual pathways—the magnocellular pathway processes luminance information, while the parvo- and koniocellular pathways also subserve color processing (for a review, see Kulikowski, 2003). The parvocellular pathway receives L and M cone input, and is sensitive to chromatic but also to luminance information, depending on the spatial scale (Reid and Shapley, 2002). Physiological studies have revealed subdivisions within the koniocellular pathway, with its middle layers involved in S-cone information processing (Hendry and Reid, 2000; Tailby et al., 2008).

The decision to define object and non-object stimuli by signals from different post-receptoral mechanisms was motivated by predictions from Bar's (2003) model that the contribution of luminance and chromatic mechanisms to object classification is not equal. In this model, luminance information significantly contributes to the speed and efficiency of object categorization, over and above the contribution of chromatic mechanisms. Initial information on shape derived from luminance detectors is rapidly transmitted through the magnocellular pathway from early visual areas to the prefrontal cortex (PFC). In the PFC, those initial cues trigger top-down facilitation of object recognition by providing the visual system with an “initial guess” on stimulus identity. Feedback from the PFC is then transmitted to the temporal cortex where it is used to facilitate bottom-up processing. The whole process results in more rapid and efficient object categorization. A functional Magnetic Resonance Imaging (fMRI) study which looked at the processing of chromatic and achromatic object contours used dynamic causal modeling to demonstrate that achromatic stimuli triggered pathways from the visual cortex to orbitofrontal cortex and from orbitofrontal cortex to fusiform gyrus, which likely reflects the top-down facilitation in object recognition by the luminance information. On the other hand, chromatic stimuli activated a direct pathway from occipital cortex to the fusiform gyrus (Kveraga et al., 2007). We therefore compared full-color and reduced-color object (or non-object) contours. Full-color stimuli contained both chromatic and luminance information [L + M, L − M, S − (L + M)]. Luminance information was absent in the reduced-color stimuli, which either excited both of the chromatic mechanisms [S − (L + M) and L−M] or only excited the S − (L + M) mechanism. An earlier ERP study by Martinovic et al. (2011) used the same paradigm as we use here. After matching stimulus contrast across conditions by use of discrimination threshold units, they found that the inclusion of luminance information results in higher accuracy and faster reaction times for object as opposed to non-object images, as well as in a reduced N1 component for object images. These results are in line with Bar's model (2003) and Kveraga et al.'s (2007) findings. As mentioned above, Valsecchi and Turatto (2007) have demonstrated that microsaccade rates are the same for isoluminant red and green stimuli and stimuli with an additional luminance edge. Through the use of two types of contrast-matched isoluminant stimuli [S − (L + M); S − (L + M) & L − M], as well as a stimulus with both chromatic and luminance information, our study can further extend the findings of Valsecchi and Turatto (2007). There are several important methodological differences between the studies. In our study, we match contrast across different types of stimuli in terms of threshold units, while Valsecchi and Turatto (2007) used stimuli that were not matched in terms of contrast. We also further divide isoluminant contrast into contrast from two chromatic cone-opponent mechanisms. The intermediate isoluminant stimulus, which excites both L − M and S − (L + M) mechanisms, is probably similar to the stimulus from Valsecchi and Turatto (2007). However, the S − (L + M) defined stimulus is dissimilar and may be particularly interesting. Methodologically, it is less likely to contain residual luminance artifacts at the edges/lines of the stimulus, as S-cone contribution to luminance is quite limited (see Ripamonti et al., 2009). Theoretically, it is also interesting because the central fovea does not contain any Scones, so S − (L + M) signals may be less salient for the generation of microsaccades than L-M cone-opponent signals.

Isolating the S − (L + M) channel enabled us to make a specific prediction, based on the fact that the central part of the fovea, about 0.3°–0.4° in size in humans, is S-cone free (Bumsted and Hendrickson, 1999). Therefore, we expect that lower fixational saccade rates should be observed for S-cone isolating stimuli but not for tGBA. If tGBA reflects mainly higher-level, object representation processes, it should not differ between S − (L + M) and intermediate isoluminant or full color stimuli. This would in turn indicate that tGBA is predominantly reflecting higher-level, cortical mechanisms of object representation. Moreover, if fixational saccades and tGBA reflect object-sensitive mechanisms, they should be enhanced for objects, as in Hassler et al. (2011). If eGBA is absent while tGBA is present, this would signify that eGBA is not a necessary and sufficient precursor to iGBA, contrary to the model of Herrmann et al. (2004b). Existing evidence already indicates that eGBA is strongly related to luminance contrast (Schadow et al., 2007). We predicted that eGBA would be absent at least from the isoluminant conditions, as our paradigm used stimuli that should not strongly engage the magnocellular pathway which has previously been related to eGBA (Fründ et al., 2007).

## Materials and methods

### Participants

Twelve healthy participants (3 males, aged 20–35 years) with normal or corrected to normal vision volunteered and gave written informed consent to take part in the eye movement experiment. All participants had normal color vision as assessed with the Cambridge Color Test (Regan et al., 1994). The study was approved by the ethics committee of the School of Psychology at the University of Aberdeen.

Eighteen healthy participants (11 males; aged 21–40 years) with normal or corrected-to-normal vision, as well as normal color vision as assessed with the Cambridge Color Test gave written informed consent to take part in the EEG experiment. One participant was subsequently removed from the sample, since more than 40% of trials were artifact-contaminated. Six further participants were removed as the ocular artifact could not be sufficiently removed from the tGBA (see section on EEG data acquisition and analysis). The participants received a small honorarium to compensate for their time. The study was approved by the ethics committee of the School of Psychology, University of Liverpool.

### Apparatus

The eye movement experiment was run on a Dell Precision PC equipped with a visual stimulus generator (Visage, Cambridge Research Systems, Ltd., Kent, UK). Stimulus presentation was controlled using Matlab (Mathworks, Natick, Massachusetts) and the stimuli were presented on a Sony GDM-520 21 inch CRT monitor. The chromatic and luminance outputs of the monitor were calibrated using the CRS calibration system (ColourCAL II, Cambridge Research Systems, Ltd., Kent, UK); the accuracy of the calibration was verified with a spectroradiometer (SpectroCal, Cambridge Research Systems, Ltd., Kent, UK). The monitor had been switched on for at least 30 min before any experiment. Participants responded via a button box (Cedrus RB-530, Cedrus Corporation, San Pedro, USA) and were seated 60 cm from the screen with their head placed in a chin rest. Binocular eye movements were recorded using an Eyelink 1000 system (SR Research, Mississauga, Ontario, Canada), which received stimulus-onset triggers from the Visage.

In the EEG experiment, an almost identical system was used for generation of stimuli and collection of responses (see Martinovic et al., 2011), with the Visage system sending triggers to a 32-electrode Biosemi Active-Two system (Biosemi, Amsterdam, Netherlands).

### Colour space

We use the DKL-color space (Derrington et al., 1984; Brainard, 1996), an extension of the MacLeod–Boynton chromaticity diagram (Macleod and Boynton, 1979), to describe the chromatic properties of our stimuli. In this space, any color is defined by modulations along three different “cardinal” axes. Along the achromatic axis, all three cone classes (L, M and S) are modulated such that the contrast is identical, that is, ΔL/L_BG_ = ΔM/M_BG_ = ΔS/S_BG_, where ΔL, ΔM, and ΔS denote the incremental cone excitations in three cone classes, respectively. L_BG_, M_BG_ and S_BG_ indicate the L-, M-, and S-cone excitations of the background. The second direction refers to a modulation along a red–green axis; modulations in this direction leave the excitation of the S cones constant (i.e., ΔS = 0), and the excitation of the L and M cones covaries as to keep their sum constant. Therefore, this axis is referred to as a “constant S-cone axis” (Kaiser and Boyton, 1996), or a “red–green isoluminant” axis (Brainard, 1996). Along the third axis, only the S cones are modulated, and ΔL = ΔM = 0. Therefore, this axis is often referred to as a “constant L & M cone” axis (Kaiser and Boyton, 1996), or as an “S-cone isoluminant” axis (Brainard, 1996) or as a “tritanopic confusion line.”

**Figure 1:**
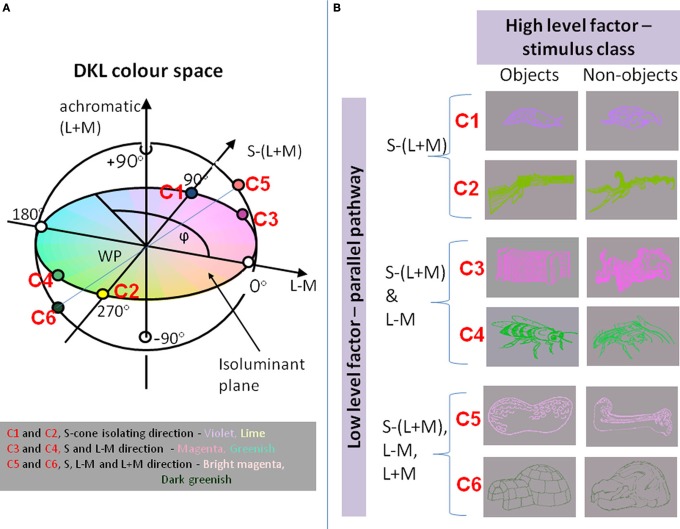
**(A)** The chromaticities of stimuli in the DKL color space. Along the achromatic axis, cone contrasts in all three cone classes vary (L + M + S). Along the L − M axis, only the difference between L- and M-cone varies, keeping L + M constant. Along the S − (L + M) axis, the difference between S cones and the sum of L and M cones varies. Colors along the S-cone-isolating line range from violet to lime; intermediate isoluminant colors range from magenta to greenish; addition of an achromatic component to the magenta and greenish stimuli results in bright magenta to dark greenish. **(B)** *Examples of stimuli*: objects and non-objects, represented in colors that excite different directions in color space.

Instead of defining the chromatic properties of a stimulus by their respective L-, M-, and S-cone modulations, the stimuli are often defined in terms of the responses of a set of hypothesized post-receptoral mechanisms that are isolated by these cardinal color modulations (Derrington et al., 1984; Brainard, 1996; Eskew et al., 1999; Wuerger et al., 2002, 2011). The three corresponding mechanisms are two cone-opponent color mechanisms and a luminance mechanism (see Figure 1A). One of the two cone-opponent mechanisms is a reddish–greenish mechanism that takes the weighted difference between the differential L- and the M-cone excitations. The second cone-opponent mechanism is a lime-violet mechanism that takes the weighted difference between the differential S-cone and the summed differential L- and M-cone excitations. The luminance mechanism sums the weighted differential L- and M-cone signals. These orthogonal mechanisms are often referred to as “L + M”, “L − M”, “S − (L + M)” (Derrington et al., 1984). For simplicity, we will define the chromatic properties of our stimuli in terms of their L,M,S cone excitations, that is, the achromatic direction as “L+M”; the reddish-greenish direction as “L−M,” and the lime-violet direction as “S.”

In the eye movement experiment, the CIE coordinates of the gray background were *x* = 0.278, *y* = 0.298 and Lum = 42.52 cd/m^2^. The endpoints of the L-M and the S directions were defined by the available monitor gamut, but constrained to be symmetric around the gray background. In terms of cone contrast, stimuli at the endpoints of the S direction were defined as follows: S increments had L and M cone contrasts 0.0 and an S- cone contrast of 0.69 while S decrements had contrasts of 0.0 for both L and M cones and −0.68 for S-cones. Increments and decrements along the L − M direction resulted in an average cone contrast in the L and M cones of 0.16 and −0.16, respectively, and 0.0 for S cone contrast.

In the EEG experiment, the CIE coordinates of the gray background were *x* = 0.296, *y* = 0.309 and Lum = 46.3 cd/m^2^. At the edge of the monitor's gamut, positive modulations along the S direction resulted in L and M cone contrasts of 0.0 and S-cone contrast of 0.89, while a negative excursion along the S direction resulted in zero contrasts for L and M cones and cone contrast of −0.89 for S-cones. The maximum incremental and decremental modulations along the L-M axis (within the available gamut) were as follows: 0.20 and -0.21 for the average LM cone contrast, and 0.0 for S cone contrast.

### Stimuli

Stimuli were taken from existing stimulus sets that contain line drawings of common objects (International Picture Naming Project with 525 pictures, Bates et al., 2003; 400 pictures from a French-language naming study, Alario and Ferrand, 1999; 152 images used in object recognition studies, Hamm and McMullen, 1998). A set of 225 objects was selected for use in the baseline threshold experiment and 168 objects were selected for use in the main classification experiment. All images represented simple, common objects from various semantic categories (for example, ship, stapler, harmonica, grasshopper, etc.; see Appendix A in (Martinovic et al., 2011) for a detailed list). Non-objects were produced by manipulating images of objects using the image distorting functions of the freely-distributed GNU Image Manipulation Programme (GIMP). After scrambling, we checked whether the resulting image adequately approximated the aspect ratio of the object it was derived from and whether it maintained the closed line structure that characterizes real objects. If not, it was edited by hand to better approximate these characteristics. Afterwards the images were converted to JPEGs and their file sizes compared. JPEG file size provides an objective estimate of visual complexity for line drawings that has been used in picture naming studies (Szekely and Bates, 2000), including the normative set provided by Bates et al. (2003). Where big discrepancies in size were present, the larger of the images were edited by hand to reduce the number of inner contours while maintaining an object-like structure. In the final stimulus set, there were no differences in visual complexity between objects and non-objects [*t*_(167)_ = 1.63, n.s.]. We also assessed low-level differences in object and non-object images by running a permutation analysis of their Fourier spectra. This analysis, using 1000 permutations, revealed that although images of objects contained more cardinally oriented lines than images of non-objects, these differences were not significant.

In the experiments, object and non-object contours were defined along three directions in DKL color space: (1) S-cone-isolating [S − (L + M)], or (2) intermediate isoluminant [S − (L + M) and L − M], or 3) a full-color stimulus with an additional achromatic component [S − (L + M); L − M; L + M], providing a luminance signal (see Figure 1). For each direction, both increments and decrements were used in order to obtain a signal that was representative for the whole direction (see Figure 1; data were collapsed across increments and decrements in the final analysis, as they did not differ significantly between each other). Thus, the stimuli either involved processing predominantly in the koniocellular pathway (S-cone-isolating contours), in both pathways capable of chromatic processing (konio- and parvocellular), or in all three visual pathways (full color images including chromatic and achromatic information: konio-, parvo- and magnocellular). The majority of the stimuli subtended a visual angle of approx. 5° × 2° (the smallest stimulus was around 3° × 1°; the biggest stimulus was around 9° × 3.5°) and were shown on a gray background. Stimulus onset was synchronized to the vertical retrace of the monitor. Stimulus presentation was balanced across the sample to control for item-specific effects: thus, across the sample, each item was presented equally often with contours defined along each of the three directions of the DKL color space.

Static random luminance noise was superimposed over the stimulus display area in the form of 3 × 3 pixel elements modulated at an RMS noise contrast of 19.5% (Ruppertsberg et al., 2003). The noise was added to each trial starting with the fixation cross preceding the stimulus presentation. The purpose of the noise was to reduce luminance-related artifactual activity which would be inevitable for isoluminant stimuli with high-frequency edges. In Martinovic et al. (2011) the same approach was used and both behavioral and ERP findings were not consistent with a luminance artifact account.

### Observer isoluminance

Individual differences in luminous efficiency may result in a small luminance artifact in the nominally isoluminant L-M signal (Wyszecki and Stiles, 2000). To control for this, prior to the experiment heterochromatic flicker photometry (HCFP; Walsh, 1958) was used to adjust the point of isoluminance for each participant.

The display alternated between two polarities of a chromatic stimulus (bluish/yellowish, magenta/greenish) at a frequency of 20 Hz. The participants adjusted the luminance of the colored stimuli in order to find a point at which the flicker was minimized. The rationale for this technique is that the chromatic system is too slow to follow fast temporal changes (flickering), while the luminance system is able to detect fast changing luminance differences. Therefore, if the perception of flicker is minimal, the difference in luminance is also minimized. Objects from the 225 threshold item set were randomly chosen as stimuli during HCFP. The procedure was repeated ten times. The lowest and highest values were then eliminated, and the mean of the remaining values taken.

### Procedure

#### Baseline experiment: threshold measurements

An initial session consisting of control measurements (Cambridge Colour Test and Heterochromatic Flicker Photometry) and the baseline psychophysical experiment was conducted with each participant, lasting one and a half hours in the eye movement experiment and 2 h in the EEG experiment.

The baseline experiment was conducted to define a common contrast metric for chromatic and luminance stimuli, as comparing responses to isoluminant and achromatic stimuli is not straightforward (Shevell and Kingdom, 2008). This difficulty can be overcome by matching the stimuli in terms of threshold units, thereby using a behavioral measure that is independent of the actual physical contrast. Such stimuli can be then used to address specific research questions regarding the role of chromatic and luminance signals in the human visual system. We took measurements of object discrimination contrast thresholds prior to the main experiment. The task required discrimination of object and non-object images taken from the same stimulus pool and was thus closely matched to the task in the main classification experiment. The reason behind this was to attempt to match effective stimulus strength (i.e., salience) for the object classification task as closely as possible. For this, a discrimination threshold with a similar task and with stimuli of similar spatio-temporal properties is much more suitable than a detection threshold, a contrast-matching threshold or a less similar discrimination threshold procedure. Cole et al. (1993) discuss the differences in neuronal populations involved in stimulus detection and in the processing of stimuli above detection threshold, with stimuli above detection threshold being encoded by a significantly larger pool of units. Zele et al. (2007) and Vassilev et al. (2009) discuss more extensively the suitability of detection threshold units for equating stimuli in terms of reaction times for rod and cone stimuli respectively.

Stimuli in the main experiment were matched in discrimination threshold units individually for each participant so that maximum possible contrast was achieved within the available gamut. This procedure was intended to ensure that any differences that emerge at suprathreshold cannot be accounted for by simple stimulus salience differences between different directions in color space. For example, a simple effect of salience would result in performance between directions in color space differing uniformly for both objects and non-objects. This was not observed in the previous study by Martinovic et al. (2011), as accuracies for non-objects remained similar across the low-level conditions, while accuracies for objects were significantly lower in the S-cone isolating condition. Due to the properties of the S − (L + M) mechanism, reductions in performance for S-cone isolating stimuli are to be expected even when attempts are made to closely match stimuli in terms of contrast (for a discussion, see O'Donell et al., 2010).

Stimulus contrast in the main experiment was adjusted toward the maximal monitor's gamut relative to discrimination thresholds in order to ensure that all stimuli were as high in contrast as possible while remaining approximately iso-salient for each individual participant. This was achieved by using multiple-of-threshold contrasts within the monitor gamut where the scaling factor was the same in all color directions. The following procedure was used to scale the stimuli: DKL radius in the direction in which the threshold was closest to the monitor's gamut was set to the value just below gamut and all the other contrasts were adjusted upwards from threshold using the scale factor calculated on the basis of this, closest-to-gamut direction. This procedure was intended to allow for an adequate signal-to-noise ratio in the EEG while maintaining equal salience along different color directions. It also allowed us to assess if behavioral measures, saccades, eGBA and tGBA relate to contrast, as different contrast level (in terms of multiple-of-threshold) was used for each participant.

**Figure 2:**
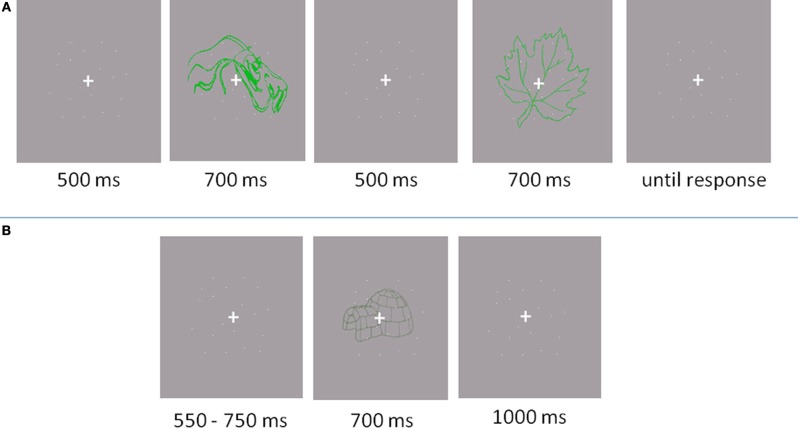
**Trail outlooks. (A)** Baseline experiment: Participants responded if the object was located in the first or the second interval. **(B)** Main experiment: Participants responded whether the presented item was an object or a non-object.

In the baseline experiment, a two-interval forced choice paradigm (2IFC) was implemented (see Figure 2A). A fixation cross (0.46 by 0.46° of visual angle) appeared in the centre of the screen for 500 ms, followed by the first item displayed for 700 ms. Subsequently, another fixation cross appeared for 500 ms, followed by the second item for another 700 ms. After the second item, participants indicated by pressing a button which of the two items represented an object. The next trial started after the response. Participants were told to give a correct answer, rather than a fast answer. Acoustic feedback was provided, indicating incorrect responses with a beep.

**Figure 3:**
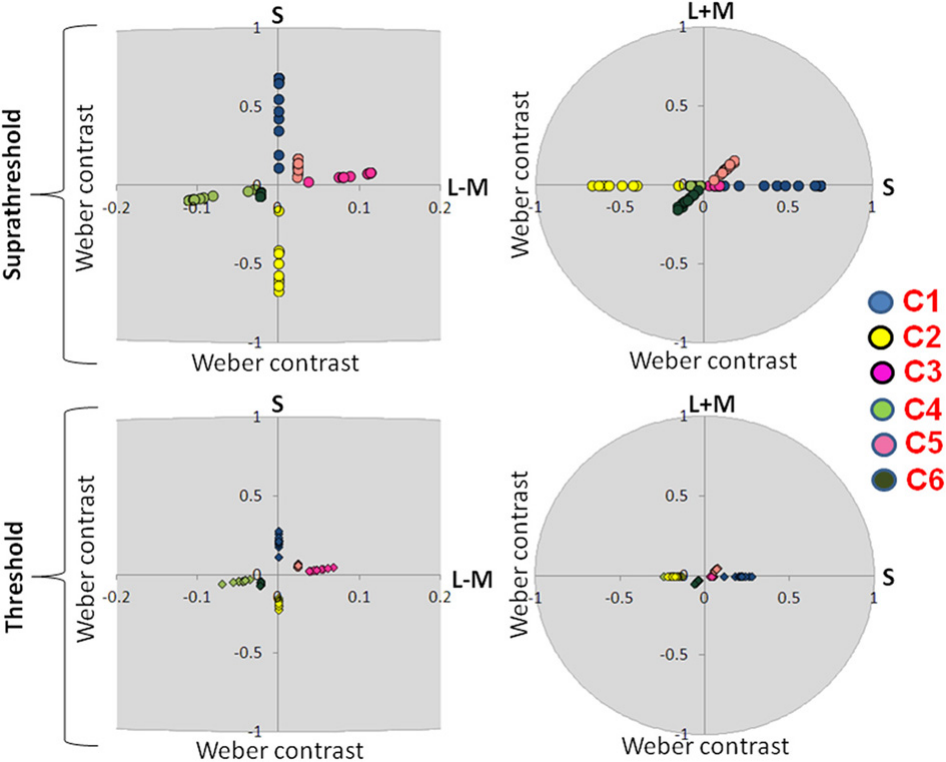
**Suprathreshold and threshold contrasts for the eye movement experiment.** Left side of the figure shows chromatic contrasts (S and L − M) while right side of the figure shows the luminance contrast in relation to S-cone contrast (S and L + M). Contrasts for each participant are represented with a single dot. C1: S-cone increment; C2: S-cone decrement; C3: intermediate isoluminant increment; C4: intermediate isoluminant decrement; C5: full-colour increment; C6: full-colour decrement.

**Figure 4:**
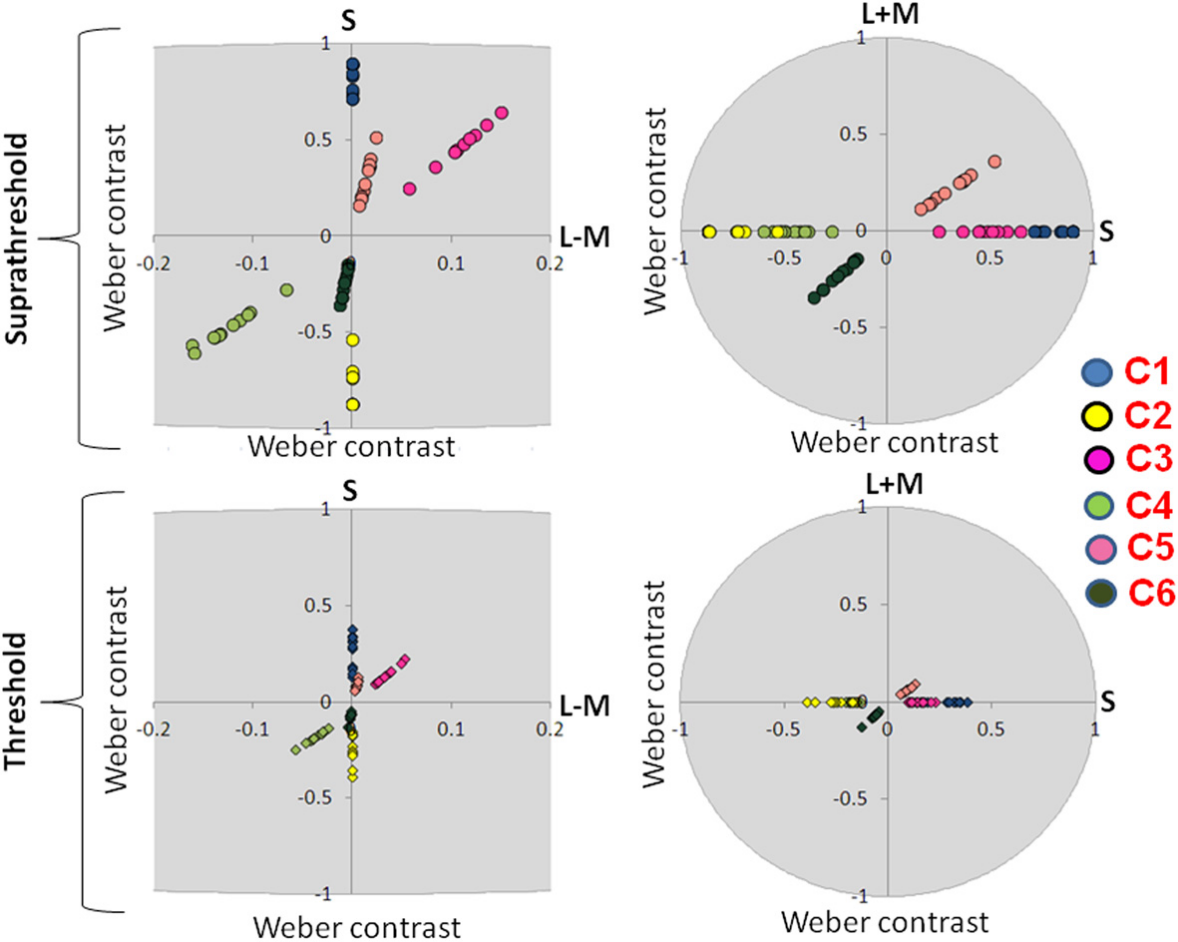
**Suprathreshold and threshold contrasts for the EEG experiment.** Left side of the figure shows chromatic contrasts (S and L − M) while right side of the figure shows the luminance contrast in relation to S-cone contrast (S and L + M). Contrasts for each participant are represented with a single dot. C1: S-cone increment; C2: S-cone decrement; C3: intermediate isoluminant increment; C4: intermediate isoluminant decrement; C5: full-colour increment; C6: full-colour decrement.

The participant's responses guided an adaptive QUEST procedure that controlled stimulus contrast (Watson and Pelli, 1983). To estimate the color contrast threshold from the relative frequency of a correct response, defined as the 81% correct point on the psychometric function, a Weibull function was fitted. In the EEG experiment, thresholds in each of the tested directions (S-cone isolating, intermediate isoluminant, full color) were measured three times for every participant; in the eye movement experiment, chromatic thresholds were measured three times while a luminance threshold was measured once and then combined with a fixed-contrast, intermediate isoluminant signal prior to scaling (see Figures 3, 4 for more detail) to create a full-colour stimulus. Differences between increment and decrement thresholds were assessed using paired *t*-tests.

#### The main experiments: EEG and eye movements

The main experiment was conducted in a separate session and lasted one and a half hours for eye movement recording and one hour for the EEG recording (see Figure 2B). First, a practice block of 20 trials was performed. The items used in the practice were not used in experimental trials. Participants were required to discriminate between drawings of familiar, nameable objects and unfamiliar, unnamable objects (non-objects). Participants were instructed to fixate the cross throughout the experiment and not to scan the presented images with their eyes. In the EEG experiment, there were four 84 trial blocks while in the eye movement experiment there were 12 blocks of 28 trials (336 trails in total). A trial started with a variable baseline period (550–750 ms) of fixation. The stimulus was then displayed for 700 ms, followed by a fixation cross displayed for 1000 ms. The participants were required to indicate whether the presented item belonged to an object or non-object category by pressing a button. Button-to-response allocation was balanced across participants. After each trial, an “X” appeared on the screen for 900 ms. The participants were advised to refrain from blinking unless the “X” was displayed.

### Behavioral data analysis

A few thresholds in the eye movement experiment were typed incorrectly into the script that computed the scale factors: for participant 2, these were the magenta and luminance decrement thresholds, for participant 4 the lime threshold and for participant 5 the lime and luminance increment thresholds. These data were left out in all subsequent analyses (behavioral and saccade rate).

The accuracies and RTs from the main experiment were analyzed. Only correct trials with RTs between 300 and 1700 ms (the maximum time allowed for responses) were used in further analyses. Median RTs for correct items were computed for each participant. Differences in accuracies and RTs between the conditions were analyzed with a 3 × 2 × 2 mixed ANOVA with the within-subject factors *direction in color space* (S- cone isolating, intermediate isoluminant, full color) and *object class* (object, non- object) and a between-subject factor of *experiment* (EEG or eye movement). Greenhouse-Geisser correction was used when necessary. *Post-hoc* paired *t*-tests with Bonferroni correction for multiple comparisons were used. Bonferroni-corrected *p*-values were adjusted by multiplying the *p* value with the number of comparisons in order to make it easier to compare them with classically used significance levels (0.05, 0.01, 0.005, 0.001).

### Eye movement recording and analysis

Recordings were performed at a sampling rate of 500 Hz. The Eyelink camera was placed on the desktop below the monitor. Participants had their head stabilized with a chin rest. The system was calibrated using the Eyelink's inbuilt 9-point calibration system. Calibration was performed at the start of the experiment and repeated between blocks if the in-built calibration check indicated that this was necessary.

Eye movements were analyzed for all correct trials using custom scripts for Matlab. Trials with saccades already detected by the Eyelink algorithm were not discarded in light of Mergenthaler and Engbert's (2010) findings; we wanted to capture not only the miniature saccades but also the somewhat larger inspection saccades. Data were segmented into epochs that included the time 500 ms before and 1500 ms after stimulus onset. Miniature saccades were detected using the Engbert and Kliegl (2003) algorithm (accessible at http://www.agnld.uni-potsdam.de/~ralf/MS). Only binocular movements were taken into further analysis. To test if the saccades in the stimulus display period (0–700 ms after stimulus onset) revealed a bimodal amplitude distribution which was found in the free-viewing study by Mergenthaler and Engbert (2010) we conducted Hartigan's unimodality test (Hartigan and Hartigan, 1985). Saccade frequencies were compared in the time window between 200 ms and 700 ms after stimulus onset for trials with correct responses. This is the time window in which the tGBA was also analyzed (see below).

Differences in fixational saccade rates between conditions were analyzed with a 3 × 2 repeated measures ANOVA with the factors *direction in color space* (S- cone isolating, intermediate isoluminant, full color) and *object class* (object, non-object). Greenhouse-Geisser correction was used when necessary. *Post-hoc* tests were performed using paired *t*-tests, with Bonferroni correction for multiple comparisons.

### EEG data acquisition and analysis

In the EEG experiment, continuous EEG was recorded from 32 locations using active Ag–AgCl electrodes (Biosemi ActiveTwo amplifier system) placed in an elastic cap. Standard locations of the international 10–20 system (Jasper, 1958) were used. In the Biosemi system the typically used “ground” electrodes in other EEG amplifiers are replaced through the use of two additional active electrodes. In the 32-electrode montage these electrodes are positioned in close proximity to the electrode Cz of the international 10–20 system: Common Mode Sense (CMS) acts as a recording reference and Driven Right Leg (DRL) serves as ground (Metting Van Rijn et al., 1990, 1991). Vertical and horizontal electrooculograms were recorded in order to exclude trials with large eye movements and blinks. EEG data processing was performed using the EEGlab toolbox (Delorme and Makeig, 2004) combined with self-written procedures running under Matlab. EEG signal was sampled at a rate of 512 Hz and epochs lasting 2000 ms were extracted, starting from 500 ms before stimulus onset and incorporating the 1500 ms after stimulus presentation. Removal of epochs with artifacts was performed using the FASTER (Fully Automated Statistical Thresholding for EEG artifact Rejection) plug-in for EEGlab (Nolan et al., 2010). The average rejection rate for artifact-contaminated trials was 22%. Trials with incorrect responses were excluded from the analysis. This left an average of 44 trials per condition. While FASTER-based artifact rejection was performed with Fz as reference, all other procedures were performed using the average reference.

The saccadic artifact was removed from the EEG using the procedure established by Keren et al. (2010). These authors derived a saccadic potential filter on the basis of data from five participants who performed an object/non-object classification task while eye movements and EEG were co-recorded. Based on Keren et al.'s (2010) suggested procedure, the eye channels were combined into a single channel referenced to the electrode Pz (radial EOG; rEOG) and data were convolved with the saccadic filter. Local peaks greater than 3.5 times the root mean square of the rEOG were identified as saccades. This threshold was selected because it produced the most similar distribution of saccades from EEG data to that observed in the actual eye movement experiment (see Figure 7A). Epochs lasting 100 ms before and after each miniature saccade were cut out. This resulted in datasets with an average of 275 epochs. Independent component analysis (ICA) was performed on these datasets using EEGlab's extended infomax algorithm (Lee et al., 1999). High-density EEG data can be considered to represent linear mixtures of activity from multiple independent generators, so ICA is intended to “unmix” them into minimally dependent source signals. When conducted on artifact-free data, ICA can reveal specific aspects of neural activity (e.g., occipital alpha-band sources; Makeig et al., 2004). It is more often used to remove ocular or muscular artifacts from EEG data since such artifacts are considered to be independent from neurally-generated activity (for a review focused on microsaccadic artifacts, see Schwartzman and Kranczioch, 2011). The major components resulting from an ICA on peri-saccadic epochs are thus likely to be those originating in the spike potential artifact. These ICAs were copied over to the complete datasets for each participant. Components that reflected typical fixational saccade activity patterns (see Keren et al., 2010) were subtracted. This resulted in a subtraction of 3 components on average (range: 0–7). Subsequently, FASTER was used again, to interpolate globally and locally contaminated channels.

Oscillatory activity in the gamma band (30–120 Hz in 4 Hz steps) was estimated using multitapers (Mitra and Pesaran, 1999) as implemented in the Fieldtrip toolbox for Matlab (Oostenveld et al., 2011). We used a fixed time window of 250 ms moved in 20 ms steps and 5 orthogonal Slepian tapers yielding a frequency smoothing of ~12 Hz. This method gives a time-varying magnitude of the signal in each frequency band leading to a time-by-frequency (TF) representation of the signal. We verified if the artifactual ocular activity was successfully removed by inspecting the time-frequency plots at all electrodes to see if the tGBA activity at frontal and eye channels was close to baseline. This led to the removal of 6 participants, with 11 participants remaining in the sample. Total GBA was analyzed in the 200–700 ms window. In order to identify the electrodes, time window and frequency range of the tGBA, mean baseline-corrected spectral activity (baseline: 200 ms prior to stimulus onset) was collapsed for all conditions together and represented in TF-plots in the 30–120 Hz range for all electrodes. Electrode sites were then selected on the basis of grand mean topographies, with maximal activity in artifact-corrected data expected at posterior sites (Keren et al., 2010; Hassler et al., 2011). Due to inter-individual differences in the induced gamma peak in the frequency domain, a maximal frequency for each participant was chosen on the basis of an average across the conditions. We used a frequency band of ±4 Hz around this peak frequency for statistical analysis. Differences in tGBA between conditions were analyzed with a 3 × 2 repeated measures ANOVA with factors *direction in color space* (S- cone isolating, intermediate isoluminant, full color) and *object class* (object, non-object). Greenhouse-Geisser correction was used when necessary. *Post-hoc* tests were performed using paired t-tests, with Bonferroni correction for multiple comparisons.

## Results

### Psychophysics: threshold measurements

Figure 3 presents scaled, suprathreshold contrasts as well as contrasts at threshold for the eye movement experiment, while Figure 4 presents these contrasts for the EEG experiment. On the left side, contrasts are plotted in the isoluminant plane (S vs. L − M); on the right side, the y-axis is the achromatic axis (L + M) and the x-axis the S-cone axis.

The scale factors in the EEG experiment ranged from 2.24 to 5.56, with the average factor being 3.46. The scale factors in the eye experiment ranged from 0.85 to 3.23, with the average factor being 2.20. These scale factors reflect the ratio of the contrast used in the experiment to that participant's threshold. The scale factors were significantly larger in the EEG experiment [*t*_(16.29)_ = 3.08, *p* = 0.007].

There were no significant differences between the threshold contrasts for increments and decrements [S − (L + M): *t*_(20)_ = 0.79, *p* = 0.44; S − (L + M) & L − M: *t*_(21)_ = −1.58, *p* = 0.13; S − (L + M) & L − M & L + M: *t*_(20)_ = −0.22, *p* = 0.83). This justified the collapsing of data across increments and decrements.

### Behavioral data: accuracy and reaction times

**Figure 5:**
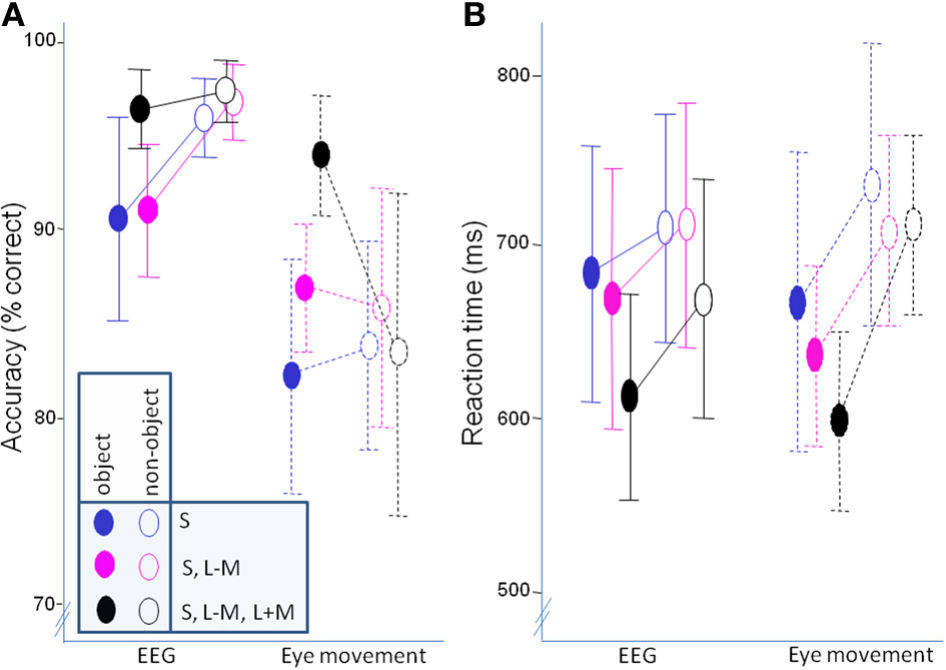
**Behavioral data. (A)** accuracy; **(B)** mean of median response times. Error bars represent 95% confidence intervals.

Figure 5A shows the accuracies while Figure 5B shows reaction times. The data was analyzed with a mixed ANOVA, as described in the behavioral data analysis section.

In accuracy, there was no overall difference between classifying objects and non-objects [*F*_(1, 21)_ = 0.06, *p* = 0.81], but there was an interaction with experiment [*F*_(1, 21)_ = 6.80, *p* = 0.02, η^2^_*p*_ = 0.25]. *Post-hoc* paired *t*-tests determined that while objects were classified less successfully than non-objects in the EEG experiment [*t*_(10)_ = −3.15, *p* = 0.02], classification accuracy did not differ in the eye movement experiment [*t*_(11)_ = 1.37, *p* = 0.80]. Independent sample *t*-tests showed that accuracy for both objects [*t*_(21)_ = 2.70, *p* = 0.013] and non-objects [*t*_(12.08)_ = 4.39, *p* = 0.001] was significantly better in the EEG experiment. There was also a main effect of direction in color space [*F*_(2, 42)_ = 7.03, *p* = 0.002, η^2^_*p*_ = 0.25], with *post-hoc* paired *t*-tests revealing worse classification of S-cone isolating stimuli than full-colour stimuli [*t*_(22)_ = −5.20, *p* = 0.0001]. On the other hand, there was no difference between the intermediate isoluminant and full-colour stimuli [*t*_(22)_ = −2.07, *p* = 0.15] and intermediate isoluminant and S-cone isolating stimuli [*t*_(22)_ = 1.40, *p* = 0.54]. This effect of direction in color space was the same for both experiments [*F*_(2, 42)_ = 0.69, *p* = 0.51]. Finally, there was an interaction between the two factors of object class and direction in color space [*F*_(1.59, 33.26)_ = 9.25, *p* = 0.001, η^2^_*p*_ = 0.31] which did not differ across experiments [*F*_(1.59, 33.26)_ = 1.75, *p* = 0.19]. Paired *t*-tests indicated that the differences between directions in color space were driven by superior performance for objects that did not contain solely S-cone signals [S-cone isolating objects vs. intermediate isoluminant objects: *t*_(22)_ = −5.22, *p* = 0.0002; S-cone isolating objects vs. full-colour objects: *t*_(22)_ = −4.70, *p* = 0.0009] with performance for intermediate isoluminant and full-colour objects and all non-objects being at a relatively similar level (Figure 5A; all *p*s > 0.1).

Reaction times were faster for objects than for non-objects [*F*_(1, 21)_ = 59.17, *p* < 0.000001, η^2^_*p*_ = 0.74], with differences between the two experiments [*F*_(2, 21)_ = 6.14, *p* = 0.02, η^2^_*p*_ = 0.23]. While there were no differences between experiments in speed of responses to objects [*t*_(21)_ = 0.49, *p* = 0.63] and non-objects [*t*_(21)_ − 0.52, *p* = 0.61], the difference between the two classes seemed to be less pronounced in the EEG experiment [*t*_(10)_ = 3.07, *p* = 0.05] than in the eye movement experiment [*t*_(11)_ = 9.09, *p* = 0.00001; see Figure 5B]. The effect of direction in color space [*F*_(2, 42)_ = 10.19, *p* = 0.0002, η^2^_*p*_ = 0.33] did not differ across experiments [*F*_(2, 42)_ = 1.11, *p* = 0.34]. The effect was somewhat different to that observed for accuracy, as *post-hoc* tests revealed that it was the speed of classification for full-colour stimuli that was most important in driving the difference, offering an advantage both when compared to S-cone isolating [*t*_(22)_ = 4.31, *p* = 0.0009] and intermediate isoluminant stimuli [*t*_(22)_ = 2.86, *p* = 0.03]. There was no difference between the two types of isoluminant stimuli [*t*_(22)_ = 1.55, *p* = 0.40]. Finally, there was also an interaction between object class and direction in color space [*F*_(2, 42)_ = 4.70, *p* = 0.01, η^2^_*p*_ = 0.18] which did not differ across experiments [*F*_(2, 42)_ = 0.62, *p* = 0.54]. The interaction was caused by the fact that the differences in RT between directions in color space occurred for full-colour vs. intermediate isoluminant objects [*t*_(22)_ = 3.51, *p* = 0.02] and full-colour vs. S-cone isolating objects [*t*_(22)_ = 4.89, *p* = 0.0006], while the speed for intermediate isoluminant vs. S-cone isolating objects and all non-objects remained similar (*p*s > 0.1).

Additionally, a Pearson correlation analysis was performed in order to examine potential relationships between behavioral responses (accuracies and mean RTs) and contrast ratios used in the experiment. A total of 12 comparisons were made and Bonferroni correction was used to correct for multiple comparisons. There was a significant correlation between contrast ratio and accuracy for S-cone isolating non-objects [*r*_(23)_ = 0.60, *p* = 0.05] and full-colour non-objects [*r*_(23)_ = 0.62, *p* = 0.02]. Other correlations were not significant: (accuracies: *r* ranging from 0.40 to 0.47; RTs: r ranging from −0.12 to −0.32; all *p*s > 0.1).

### Fixational saccades

**Figure 6:**
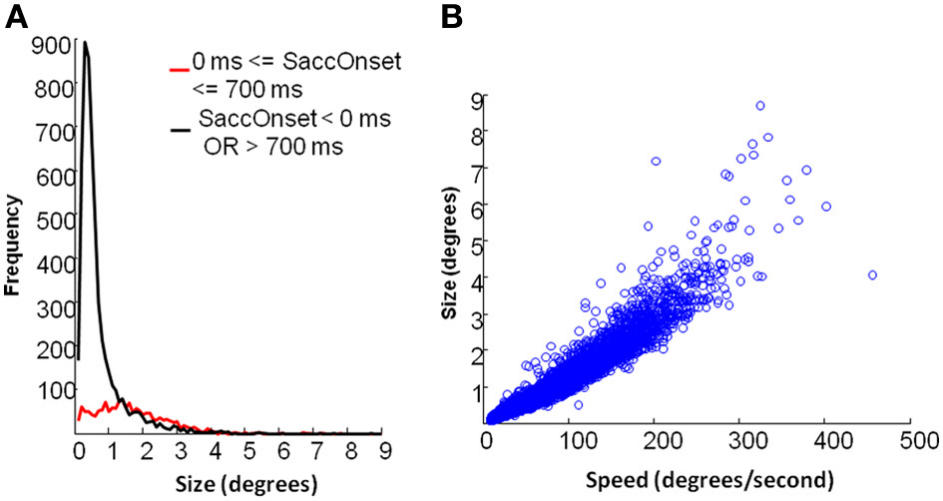
**Eye movement data: saccade properties. (A)** Distribution of saccades by size. Black line depicts saccades during fixation cross and red line depicts saccades during picture presentation. **(B)** main sequence relation between speed and size of saccades for the period of picture presentation.

As shown in Figure 6A, fixational saccades during picture presentation (0–700 ms after stimulus onset) included a broad range of differently-sized saccades. On the contrary, very small saccades were dominant during periods when the fixation cross was displayed. In our analysis, the fixation cross period involved 500 ms of fixation prior to the stimulus onset and 800 ms after stimulus offset. Figure 6B indicates that fixational saccades during picture presentation showed a linear relation between size and speed (also known as the main sequence). Hartigan's unimodality test showed that the distribution of saccades during picture presentation was not multi-modal (*p* = 0.59). Therefore, we analyzed the frequencies of saccades in this period irrespective of their size.

**Figure 7:**
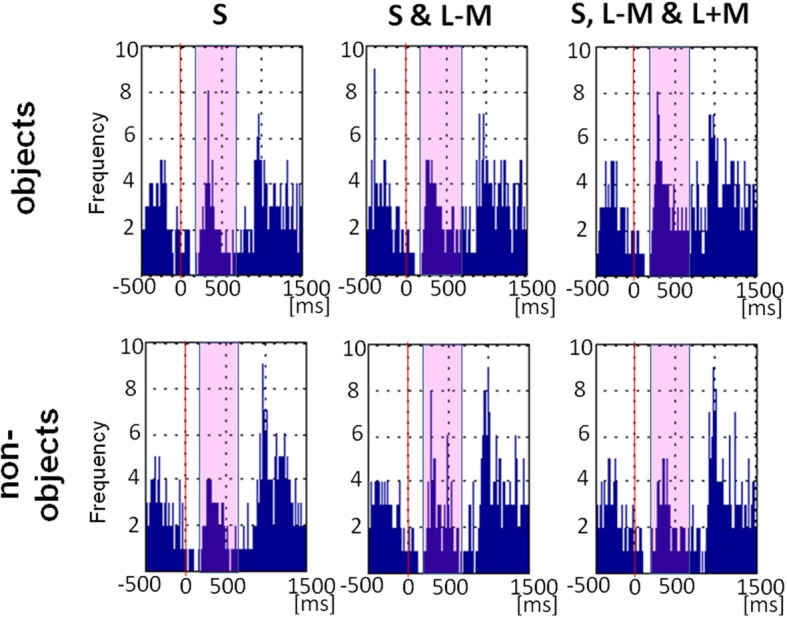
**Eye movement data: saccade rates across time.** Frequency plot of all fixational saccades in the period including −500 ms before picture onset and 1500 ms after picture onset. Solid red line indicates stimulus onset and the magenta rectangle highlights the period 200–700 ms post-stimulus which was the main focus of our analysis.

Figure 7 shows the plot of fixational saccade rates across time. Fixational saccades drop substantially 100–150 ms after picture presentation, peaking from approx. 200 to 500ms. There was a main effect of object class [*F*_(1, 11)_ = 4.78, *p* = 0.05, η^2^_*p*_ = 0.30], with more fixational saccades for objects (*M* = 22.17, *SE* = 5.46) than for non-objects (*M* = 18.36, *SE* = 4.49). There was a main effect of direction in color space [*F*_(2, 22)_ = 6.77, *p* = 0.005, η^2^_*p*_ = 0.38), indicating that fixational saccade rates differed across the three color directions, while there was no significant interaction between the factors direction in color space and objecthood [*F*_(2, 22)_ = 1.84, *p* = 0.18]. *Post-hoc* tests revealed that the difference between color directions was driven by higher saccadic rates for full stimuli (*M* = 25.00, *SE* = 6.00) than for S-cone isolating stimuli (*M* = 15.29, *SE* = 3.83; *p* = 0.03), with intermediate isoluminant stimuli (*M* = 20.50, *SE* = 5.39) not being different from full stimuli (*p* = 0.13) or from S-cone isolating stimuli (*p* = 0.25).

Again, we performed a Pearson correlation analysis to examine the relationship between behavioral responses (accuracies and mean RTs), contrast ratios, and rates of fixational saccades in the period between 200 and 700 ms. A total of 18 comparisons were made and Bonferroni correction was used to correct for multiple comparisons. No significant correlations were found: (accuracies: *r* ranging from 0.03 to 0.43; RTs: *r* ranging from −0.55 to −0.35; contrast ratios: *r* = ranging from 0.32 to 0.63; all *p*s > 0.1).

### Gamma-band activity

Successful removal of miniature saccade artifacts using the saccadic potential filter (Keren et al., 2010) was possible in 11 out of 17 participants. Visual inspection revealed that the remaining 6 participants still had relatively high tGBA at ocular and frontal channels after artifact removal. The relatively low efficiency of artifact removal could be due to the reduced rate of fixational saccades (see fixational saccade results) in our study when compared to Yuval-Greenberg et al. (2008) and Keren et al. (2010). A lower saccade rate reduces the amount of data that is fed into the ICA which adversely impacts the quality of the artifact removal. In our eye movement experiment, the number of fixational saccades was found to vary vastly between participants, with 6 out of 12 participants having a total of 80 or less fixational saccades during the 200–700 ms period after picture presentation while other participants had between 123 and 291 saccades in this period (large individual differences in fixational saccade rates were also reported by Makin et al., 2011). The number of participants with relatively low saccade rates approximately corresponds to the number of participants in the EEG study (6 out of 18) in whom artifact removal was not successful. An independent *t*-test revealed that the number of 'saccades' detected with the saccadic potential filter was lower in the 6 rejected participants (*M*_reject_ = 262, *SD*_reject_ = 25; *M*_sample_ = 290, *SD*_sample_ = 28; *t*_(15)_ = 2.09, *p* = 0.05), indicating that it could indeed be that lower saccade rates in those participants may have led to an artifact which could not be effectively removed with the ICA procedure. It is important to note that the one participant in whom there were no components that appeared to correspond to the known topographical and temporal properties of the artifact was not removed from the sample, since tGBA did not show the typical artificial pattern. Therefore, we assume that he maintained fixation successfully, while the rejected participants probably made fewer and/or smaller fixational saccades that did not allow their proper identification with the Keren et al. (2010) method.

**Figure 8:**
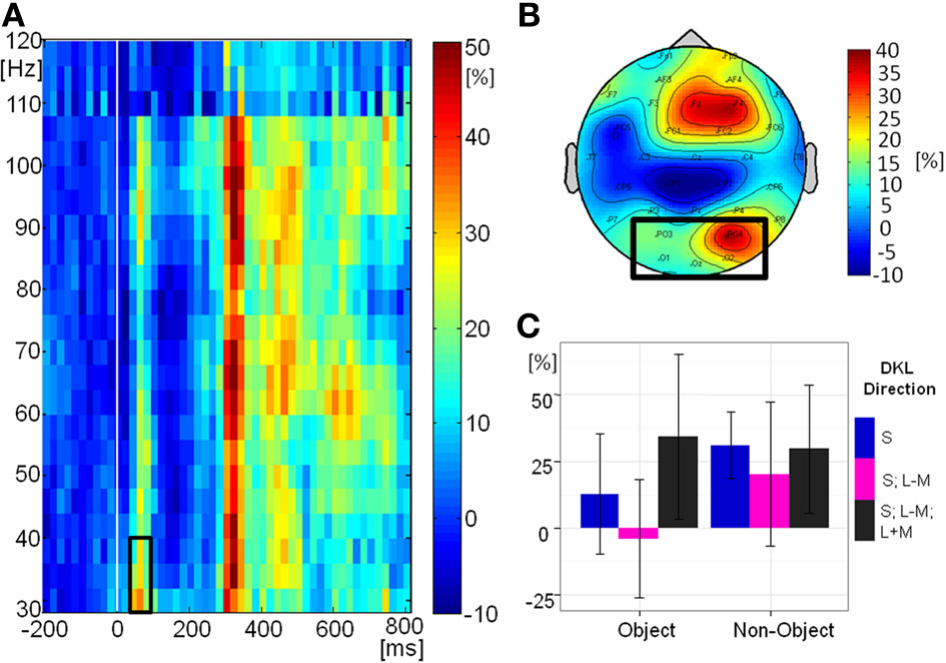
**Evoked GBA. (A)** Grand mean baseline-corrected TF-plot averaged at the regional mean sites (see **panel B**) across all conditions. Box indicates the time window for statistical analysis. **(B)** Grand mean amplitude-map (average across all conditions) for activity within the black box in Panel **A**). Box indicates electrode sites included in the regional mean. **(C)** Bar plot of amplitudes of evoked GBA for each condition at the regional mean during the selected time window, with 95% confidence interval bars.

Figure 8A shows the grand-mean time-course of the eGBA at posterior electrodes, Figure 8B shows the topography and Figure 8C shows the relative change in signal power from baseline in the analyzed time-frequency window. There was no significant effect of object class on eGBA relative power [*F*_(1, 10)_ = 2.76, *p* = 0.1). There was a significant effect of direction in color space [*F*_(2, 20)_ = 5.00, *p* = 0.02, η^2^_*p*_ = 0.33). *Post-hoc t*-tests showed that the eGBA relative power was significantly lower for intermediate isoluminant stimuli than for full-colour stimuli (*p* = 0.04); no other comparisons were significant (all *p*-values > 0.1). There was no interaction between object class and direction in color space [*F*_(2, 20)_ = 1.55, *p* = 0.2]. Evoked GBA was significant compared to baseline only in the S-cone isolating non-object condition (*p* = 0.002; all other *p*s > 0.1).

**Figure 9:**
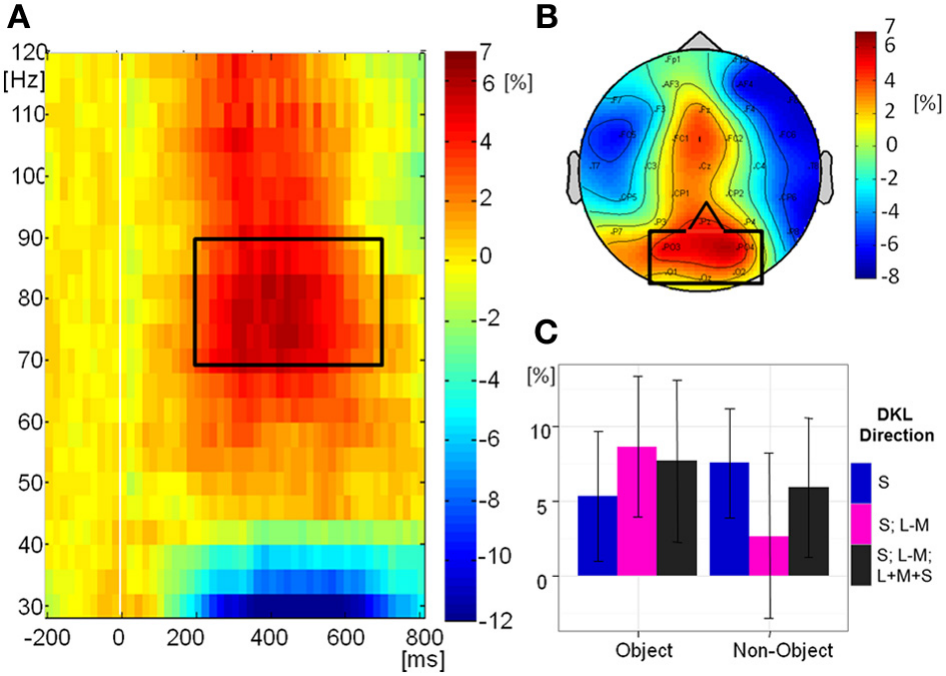
**Total GBA. (A)** Grand mean baseline-corrected TF-plot averaged at the regional mean sites (see **panel B**) across all conditions. Box indicates the time window for statistical analysis. **(B)** Grand mean amplitude-map (average across all conditions) for activity within the black box in **panel A**). Box indicates electrode sites included in the regional mean. **(C)** Bar plot of amplitudes of total GBA for each condition at the regional mean during the selected time window, with 95% confidence interval bars.

Figure 9A shows the grand-mean time-course of the tGBA at posterior electrodes, Figure 9B shows the topography while Figure 9C shows the relative change in power from baseline in the analyzed time-frequency window. There was no significant effect of object class [*F*_(1, 10)_ = 1.38, *p* = 0.3] or direction in color space [*F*_(2, 20)_ = 0.11, *p* = 0.9] on tGBA relative power. There was a significant interaction between object class and direction in color space [*F*_(2, 20)_ = 3.77, *p* = 0.04, η^2^_*p*_ = 0.27]. While *post-hoc* comparisons were not significant, it would appear from the graph (Figure 9C) that relative power is higher for intermediate isoluminant objects than for intermediate isoluminant non-objects, while for S-cone isolating and full-colour stimuli the relative powers are roughly similar for objects and non-objects. Total GBA was significant compared to baseline in the S-cone isolating non-object condition (*p* = 0.006) and the intermediate isoluminant object condition (*p* = 0.01), with a trend toward significance for the full-colour object condition (*p* = 0.06; all other *p*s > 0.1).

As before, we performed a Pearson correlation analysis in order to establish whether there are relations between behavioral responses (accuracies and mean RTs) and contrast ratios used in the experiment, on one hand, and tGBA in the period between 200 and 700 ms, on the other hand. As a total of 18 comparisons were made, Bonferroni correction was used. There was a trend for total GBA for intermediate isoluminant non-objects to correlate with speed of responding to these non-objects [*r*_(11)_ = −0.79, *p* = 0.07]. Other correlations were not significant: (accuracies: *r* ranging from -0.37 to 0.77; RTs: *r* ranging from −0.48 to 0.71; contrast ratios: *r* ranging from −0.21 to 0.34; all *p*s > 0.1).

The same type of analysis was performed for eGBA but no significant correlations were found (accuracies: *r* ranging from −0.59 to 0.73; RTs: *r* ranging from −0.54 to −0.02; contrast ratios: *r* ranging from −0.53 to 0.47; all *p*s > 0.1).

## Discussion

We investigated modulations of behavioral responses, fixational saccades and gamma-band activity by low- and high-level factors in an object classification task. Stimuli were defined along different directions in cardinal color space so that they differentially excited distinct post-receptoral mechanisms, with contrasts matched in terms of discrimination thresholds. This provided a controlled low-level manipulation, while stimulus class (object or non-object) provided a high-level manipulation. In both the eye movement and the EEG experiments, behavioral performance was the fastest for full-colour objects and least accurate for S-cone isolating objects, with performance for non-objects remaining similar across all directions in color space. The stimulus contrasts were somewhat higher in the EEG experiment, but in the analysis, the experiment factor only interacted with object class, with an accuracy advantage for classifying objects in the EEG experiment but not in the eye movement experiment, and a less pronounced reaction time advantage for objects in the EEG experiment. Performance for S-cone isolating and full-colour non-objects was also correlated with contrast. Therefore, lower contrast seems to have a more adverse effect on performance for non-objects. Fixational saccade rates 200–700 ms after stimulus onset depended on low and high-level factors independently, being higher for full-colour stimuli and for objects. Evoked GBA was fairly low and its amplitude was modulated by low-level factors only. In contrast, artifact-free, low-amplitude sustained tGBA that lasted approximately 200–700 ms was dependent on both low and high-level factors.

The behavioral results extend the pattern from the previously conducted EEG experiment (Martinovic et al., 2011): performance for objects differs across the directions in color space, while performance for non-objects remains steady. Differences between the two experiments were observed only in terms of responses to stimulus class, with performance in the EEG experiment being more accurate overall, with less pronounced differences between the two stimulus classes in terms of reaction times. The most substantial difference between the two experiments was in terms of maximal achievable contrasts, which resulted in significantly higher contrast ratios in the EEG experiment. As accuracy was related to contrast ratios for two out of three non-object conditions, this would imply that non-object performance is more driven by contrast. This finding emphasizes the importance of low-level signals in driving task performance: although the contrasts were set to various multiple-of-threshold levels, these levels may have been close enough to threshold to still enact an influence on accuracy rates. Ceiling effects that are commonly observed in object classification experiments (e.g., Gruber and Müller, 2005; Busch et al., 2006) were not reached, except perhaps for the full combination objects and non-objects in the EEG experiment.

Mergenthaler and Engbert (2010) demonstrated that in a free viewing task saccades are distributed bimodally, with those below 0.4° less numerous and predominantly around 0.1° in size, and those above 0.4° much more numerous and mostly around 10° in size (their stimulus was presented full screen). On the contrary, in their fixational task, saccades were distributed unimodally with a peak around 0.5° and the vast majority of saccades smaller than 1°. In our study, fixational saccades observed before and after stimulus presentation match the distribution of saccades in Mergenthaler and Engbert's fixation task. However, we find that saccades during picture presentation contained a significant proportion of larger saccades (>1°) when compared to saccades made during periods when only the fixation cross was presented. We did not observe a bi-modal distribution. In fact, with saccades over 1° prominent in our data, it could be that an onset of a complex stimulus within the fixation area preferentially elicits inspection saccades and perhaps even voluntary, exploratory saccades. This suggestion is in line with a recent study by Otero-Millan et al. (2013), which suggests that fixation and exploration behaviors are not in fact different, opposing phenomena, but can rather be placed on the extremes of the same continuum. In their study, Otero-Milan et al. presented observers with scenes of varying sizes and found that as the scenes decreased in size, so did the size of produced saccades. Otero-Milan et al. report that in a free-viewing task the saccade magnitude distribution ranged from 0.1 to 10 deg for stimuli sized between 4 and 8 deg in width, with less saccades for the blank scenes than for natural scenes. In line with this finding, it is perhaps not surprising to observe more inspection saccades in our experiment, as participants are asked to classify images containing relatively low-contrast, task-relevant visual content—however, this suggestion warrants further investigation.

Otero-Millan et al. (2008) demonstrated that high-level modulations of microsaccades can occur. In our study, fixational saccade rates 200–700 ms after stimulus onset were enhanced for objects as opposed to non-objects, in line with Hassler et al. (2011) and Yuval-Greenberg et al. (2008). Modulations of microsaccades by low-level factors observed in our experiment extend previous findings. Valsecchi and Turatto (2007) demonstrated that the characteristic microsaccadic signature rate was observable for isoluminant red-green stimuli and did not differ significantly from the saccades elicited by stimuli defined with a further luminance component. If the superior colliculus is “color blind”, as Marrocco and Li's (1977) findings are often taken to suggest, then Valsecchi and Turatto's (2007) results suggest that cortical areas responsive to color are involved in microsaccades. Here we demonstrate that S-cone isolating contours result in fewer fixational saccades compared to full-colour stimuli, without finding a significant difference for the intermediate isoluminant stimulus. While S-cones do not project directly to the superior layers of the superior colliculus, S-cone elicited neural responses have been reported to be as fast as L − M elicited responses at the level of its intermediate layers (White et al., 2009), indicating cortico-tectal loops of similar timing (but see also Tailby et al., 2012). Fixational behavior is related to foveating the target of interest, and our findings support the suggestion that fixational saccades are highly related to the acquisition of fine spatial details during foveal processing (Ko et al., 2010) and play a very important part in edge detection (Kuang et al., 2012). This is also in line with Otero-Millan et al. (2008), who reported increases in fixational saccades rates in a visual search task in those parts of the image that contained the targets.

As mentioned in the introduction, the central part of the fovea (approx. 0.3°–0.4°) does not contain S-cones. Thus, S-cones could perhaps play a less important role in driving exploratory saccades that are coupled with foveal processing strategies. Our results on fixational oculomotor behavior complement findings on voluntary saccades driven by S-cone isolating stimuli, with absence of overt but not covert inhibition of return (Sumner et al., 2002, 2004) already reported. Further, visual search is less efficient for stimuli that differ from other elements in the search array only in S-cone increment contrast (Lindsey et al., 2010). The low-level and high-level influences on fixational saccades were independent of each other, implying two separate control systems. Fixational saccade rates were reduced for S-cone isolating contours compared to full color contours which parallels the effect observed for accuracy. However, they did not correlate with contrast or performance measures, which suggests that they did not make a particularly strong contribution to efficient task performance.

An alternative account of our behavioral and fixational saccade findings would be that the multiple-of-discrimination-threshold approach did not appropriately match contrasts between different directions in color space, S − (L + M) stimuli being particularly adversely affected. This would have led to a reduction in both performance and fixational saccades. There are several arguments against this interpretation. A contrast mismatch would have led to general differences in saliency, thus similar patterns of results should be expected for objects and non-objects. However, we observed an interaction between the two factors in the analysis of accuracy rates and reaction times, with performance differences between directions in color space emerging for objects but not for non-objects. The overall levels of accuracy were, however, relatively low. Although stimuli were displayed at on average 2–3.5 times threshold, performance in the majority of conditions did not reach ceiling, ranging from around 83% correct to around 97% correct (see Figure 5A). Thresholds were measured for discriminating objects from non-objects in a 2IFC paradigm, while the main experiments use single-trial discrimination of images. Transition to a one-interval forced choice (1IFC) would lead to a decrease of performance equivalent to $\sqrt{2}$ times 2IFC threshold (Kingdom and Prins, 2010). While the performance decrease for S-cone isolating stimuli in the eye movement experiment can be approximated in this fashion on the basis of units-of-threshold, this is not the case for full-colour stimuli, in which performance for objects is far superior than what would be predicted simply on the basis of 2IFC-to-1IFC performance transition (see Figure 5A). As discussed previously, differences between object and non-object performance and their relations to suprathreshold contrast are an important result of this study. There is, however, one more potential issue that could emerge due to the transition between 2IFC and 1IFC: the single-trial task has the problem of being “criterion-dependent” (for a detailed elaboration, see Kingdom and Prins, 2010). There is a risk that the criterion-free 2IFC is not suitable for equating contrasts for single-trial yes/no tasks if the transition to a single trial also introduces a large bias. This can cause differences in accuracy, as the biased category would receive near-ceiling accuracy while the opposite category would have much lower accuracy rates. In a recent study, we have found that single-trial classification of line drawing objects and non-objects, such as those used in this study, does not introduce biases and results in similar sensitivity across different mechanisms and their combinations for stimuli at threshold (Martinovic et al., 2013). In addition to that, inspection of Figure 5A demonstrates that ceiling effects were not consistently reached for objects or non-objects, which is another argument against a large bias for any of the two categories in our multiple-of-threshold stimuli.

In the EEG, we observed low levels of gamma-band activity, with above baseline eGBA in 1 of 6 conditions and above baseline tGBA in 2 out of 6 conditions. Evoked GBA was related to iGBA in a causal fashion by Herrmann et al. (2004b) and in the S-cone isolating non-object condition in our study both responses are indeed above baseline. However, this is not the case for the other condition with significant tGBA. All previous studies with visual objects resulted in a robust, high-amplitude eGBA response, followed by a small-amplitude iGBA (see e.g., Busch et al., 2006; Fründ et al., 2008; Martinovic et al., 2008a,b). Although our data provides some support that the two responses are likely to occur together, it also partly runs contrary to Herrmann et al.'s (2004b) memory match and utilization model, since eGBA does not always precede tGBA. The modulations of evoked and total GBA in our study also dissociate, with eGBA being influenced by low-level factors and tGBA showing a combined low and high-level modulation. The tGBA effect seems to be driven by the difference between intermediate isoluminant objects and non-objects (see Figure 9C). Larger tGBA relative power for intermediate isoluminant non-objects also showed a tendency to be associated with faster responses, which indicates that tGBA 200–700 ms post-stimulus onset might relate to task performance. However, the fact that the signals are weak and thus likely to be noisy makes these effects very difficult to interpret and necessitates a replication.

Furthermore, around one third of participants (6 out of 17) were rejected due to inadequate ocular artifact removal from tGBA. It can be argued that this was because tGBA and fixational saccades are intrinsically coupled, and therefore it is problematic to remove ocular artifacts without removing cortical-only signal. However, Craddock et al. (2013) have already used the Keren et al. (2010) approach successfully to remove ocular artifacts and reveal underlying tGBA. Therefore, we presume that artifact rejection has failed on those participants due to the fact that they made smaller numbers of fixational saccades. The ICA approach relies predominantly on the quality and amount of the initial input (Groppe et al., 2009)—in other words, if there were not enough fixational movements to successfully train the algorithm, this would adversely affect the artifact removal procedure. We did indeed have fewer peri-saccadic trials to subject to the ICA for these rejected participants than for the rest of the sample. We consider this to be due to the relatively low levels of saccades elicited by our stimuli. Poletti and Rucci (2010) suggested that the required precision of fixation has a great contribution to the miniature saccade rate's modulation and our experiments had a fixation cross superimposed over the stimulus, unlike Hassler et al. (2011) and Yuval-Greenberg et al. (2008). The number of miniature saccades decreases as the fixation target gets bigger (McCamy et al., 2013), but we used a relatively small fixation cross. Perhaps even more importantly, the stimuli were of low contrast when compared to those usually used in object recognition studies, which is likely to result in fewer microsaccades (Cui et al., 2009). When considering artifact removal efficiency in terms of the fixational saccade findings of Cui et al. (2009), one should also consider the difference in suprathreshold contrast between experiments. Since contrast was lower for participants in the eye movement experiment, the number of saccades in the EEG experiment was likely to have been larger, but in spite of that artifact removal proved to be problematic in a large number of participants.

The number of analyzed trials per condition is also important in achieving adequate signal-to-noise ratio when studying small amplitude EEG components. In our study, the number of analyzed trials does not differ much to studies on GBA prior to the publication of Yuval-Greenberg et al.'s paper in 2008. For example, an average of 44 trials in this experiment compares to 47 trials in Martinovic et al. (2007). However, since the removal of ocular artifact reduces overall amplitude, the number of trials could have posed an additional problem for obtaining adequate signal-to-noise ratio in the tGBA window (Jerbi et al., 2009). Insufficient number of trials would have had adverse effect on the gamma-activity levels. However, there are inherent limitations when working with meaningful, nameable stimulus sets. We used 225 images for threshold 2IFC measurements and 168 images for the single-trial main experiments, compiled from a range of existing stimulus sets. It is difficult to include more images without having pictures of familiar objects that look overly similar, introducing undesirable memory effects, or including images of relatively unfamiliar objects or objects from non-canonical views which pose their own recognition challenges. Recent studies with meaningful, nameable object stimuli used 100 stimuli per condition (Hassler et al., 2013) and 74 stimuli per condition (Craddock et al., 2013), which is higher than the 56 stimuli per condition in this study. A study with a larger number of stimuli, utilizing matched-contrast isoluminant conditions, would be needed before a firm conclusion could be made that isoluminant line-drawing stimuli are not suitable for eliciting GBA in general.

Comparison of fixational saccade findings and GBA findings is complicated by the fact that they were conducted on two samples which differed in contrast levels at which the stimuli were displayed. However, in terms of performance, between-experiment differences concerned only object-class, indicating that lower contrast has a more adverse effect on performance for non-objects. The important finding that performance for line-drawings of objects is more contrast-invariant will need to be replicated with other stimulus materials (e.g., outlines, line fragmented stimuli, Gaborised stimuli). The main importance of this study is that it shows for the first time that peaks in saccade rate around 200–700 ms after stimulus onset are attenuated for S-cone isolating stimuli when compared to full-colour stimuli and that fixational saccades exhibit independent low and high-level effects, in line with Engbert's (2012) recent model. No relations with behavioral performance or contrast were found. On the other hand, eGBA 50–150 ms after stimulus onset depends on low-level factors and tGBA 200–700 ms after stimulus onset depends on both low and high-level factors, although both are of very low amplitude in this particular paradigm. Both fixational saccades and GBA therefore appear to be useful markers of visual processes involved in object recognition and classification, although studies with isoluminant and/or low contrast luminance stimuli may not be ideal for eliciting robust GBA. We conclude that cortical loops involved in the processing of objects are preferentially excited by stimuli that contain achromatic information. Their activation can lead to relatively early exploratory eye movements even for foveally-presented stimuli.

### Conflict of interest statement

The authors declare that the research was conducted in the absence of any commercial or financial relationships that could be construed as a potential conflict of interest.
